# Nanoplastics: Status and Knowledge Gaps in the Finalization of Environmental Risk Assessments

**DOI:** 10.3390/toxics10050270

**Published:** 2022-05-23

**Authors:** Andrea Masseroni, Cristiana Rizzi, Chiara Urani, Sara Villa

**Affiliations:** Department of Earth and Environmental Sciences DISAT, University of Milano-Bicocca, Piazza della Scienza 1, 20126 Milano, Italy; a.masseroni2@campus.unimib.it (A.M.); chiara.urani@unimib.it (C.U.); sara.villa@unimib.it (S.V.)

**Keywords:** nanoplastics, environmental risk assessment, effects, exposure, polystyrene

## Abstract

Nanoplastics (NPs) are particles ranging in size between 1 and 1000 nm, and they are a form of environmental contaminant of great ecotoxicological concern. Although NPs are widespread across ecosystems, they have only recently garnered growing attention from both the scientific community and regulatory bodies. The present study reviews scientific literature related to the exposure and effects of NPs and identifies research gaps that impede the finalization of related environmental risk assessments (ERAs). Approximately 80 articles published between 2012 and 2021 were considered. Very few studies (eight articles) focused on the presence of NPs in biotic matrices, whereas the majority of the studies (62 articles) assessed the lethal and sublethal effects of NPs on aquatic and terrestrial organisms. Whilst many studies focused on nude NPs, only a few considered their association with different aggregates. Amongst NPs, the effects of polystyrene are the most extensively reported to date. Moreover, the effects of NPs on aquatic organisms are better characterized than those on terrestrial organisms. NP concentrations detected in water were close to or even higher than the sublethal levels for organisms. An ERA framework specifically tailored to NPs is proposed.

## 1. Introduction

### 1.1. The Plastic Era

The term ‘plastics’ is commonly used to describe a wide range of synthetic and semi-synthetic materials, with over 5300 existing polymers [1]. According to the International Organization for Standardization (ISO), plastics are defined as ‘materials that contain, as an essential ingredient, a high-molecular-weight polymer and that, at some stage in its processing into finished products, can be shaped by flow’ [2]. According to the International Union of Pure and Applied Chemistry (IUPAC), plastics are ‘polymeric materials that may contain other substances to improve performance and/or reduce costs’ [3].

**Figure 1:**
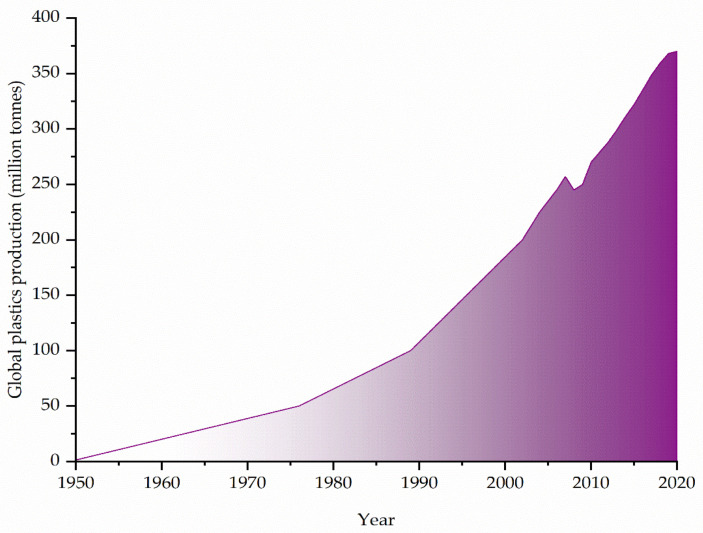
Global plastic production from 1950 to 2020 [5].

Although it is considered a ‘modern’ material, the history of plastic began in the 19th century, with the patent for the first semi-synthetic plastic material by Alexander Parkes. In 1909, the first resin of synthetic origin was produced in the laboratory by the Belgian chemist Leo Baekeland, who patented it under the name of Bakelite. The discovery of this material, which for many years was the most widespread and used plastic, was followed by the invention of a process for the production of polyvinyl chloride (PVC) in 1912 and cellophane in 1913. Between the two World Wars, the plastics industry underwent a remarkable expansion with the invention of nylon, polystyrene (PS), polyethylene terephthalate (PET) (1938), and polypropylene (PP) (1951) as well as the discovery of stereospecific polymerization to form isotactic polypropylene (1954), for which Giulio Natta and Karl Ziegler jointly won the Nobel Prize in Chemistry in 1963 [1,4]. Since then, plastic has become an irreplaceable material in everyday life. Since the 1950s, global plastic production has grown rapidly, as shown in Figure 1. In 2020, nearly 370 million tons of plastic were produced globally, of which 55 million tons, due to the strong relationship between plastic production and demand, were produced in Europe alone. Plastic materials are widely used because of their excellent physical and chemical properties (e.g., malleability, durability, lightness, corrosion resistance, and electrical insulation), which render them ideal for a wide range of applications [5].

The most widely used plastic polymers (with an average global production of more than 5% of the total) are polypropylene (PP), polyethylene (PE), PVC, PET, polyurethane (PUR), and polystyrene/expandable polystyrene (PS/EPS) (Table 1). These plastics are principally employed in food packaging, automotive parts, and household goods. PVC and PUR are mainly used in the building and construction sector and in the automotive industry, whilst PET is principally utilized in drink bottles and textile applications [6]. PS is used in packaging and insulation materials. Together, these polymers represented over 80% of all plastics used in Europe in 2020. Despite being designed to last for a long time, plastics are used only briefly, particularly in the packaging sector. According to the World Bank estimate, in 2016, the amount of plastic waste generated was 242 million metric tons in the world, of which 57 million metric tons were produced in Asia, 45 million metric tons in overall Europe, and 35 million metric tons in North America. As the global production in the same year was 336 million metric tons, approximately 70% of the produced plastic was not recycled [4] and possibly entered the environment.

### 1.2. Characteristics of Plastic Debris Present in the Environment

The physical and chemical properties of plastics make them a unique and excellent material. However, these properties do not allow decomposing organisms to digest plastic polymers (e.g., because of their rigid structure and high molecular weight) [7]. Nonetheless, once released into the environment, plastic materials are exposed to different environmental reactions (through physical fragmentation or chemical degradation pathways), which decrease their molecular weight and cause chemical changes [8]. Following these processes, plastics are fragmented into smaller particles [1,8]. The size of plastic debris plays a fundamental role in ecological dynamics, representing one of the main factors driving the environmental interaction between plastics and biota [9,10,11]. As pointed out by Mitrano et al. [12], size contributes to the characterisation of the physical and chemical properties of plastics, thus affecting their mobility, environmental and biodistribution, and interaction with organisms.

**Figure 2:**
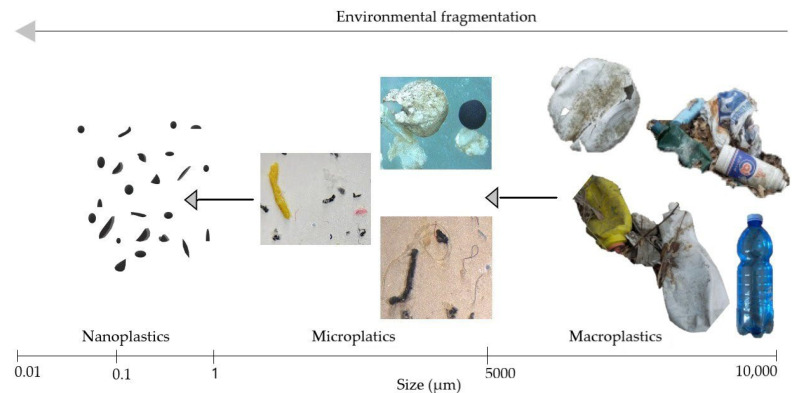
Graphical representation of plastic debris of macro, micro, and nano size (please note that the images representing plastic particles are not in scale).

The classification of plastic debris based on size presents ambiguous terminology owing to the lack of universal definitions for different size ranges [9,13]. Typically, the classification of microplastics (MPs) as particles <5 mm is accepted [14]: the upper 5 mm limit is biologically significant because a wide range of small particles of this size (<5 mm) can be ingested by organisms [1]. However, the upper size limit of nanoplastics (NPs) remains controversial, and an arbitrary size cut-off of 100 or 1000 nm is selected. The upper 100 nm limit is based on the definition of nanomaterials proposed by the US National Nanotechnology Initiative [11]. NPs present different properties from classic nanomaterials because they are not intentionally produced in the nanometric size range. Furthermore, both in terms of methodologies required for their detection and their properties and toxic effects, plastic fragments are significantly different when they cross the size scale from micrometers to nanometers [11]. As proposed by Gigault et al. [13], NPs should, therefore, be considered as ‘the plastic particles that present colloidal behaviour within the size range of 1 to 1000 nm’. According to this classification, which covers the entire nanometric size range in the definition of NPs, plastic particles are defined as macroplastics (>5 mm), MPs (1–5 mm), and NPs (1–1000 nm) (Figure 2).

### 1.3. Environmental Risk Assessment (ERA) of Plastic Debris

ERA is a rigorous process for quantifying how likely an ecosystem is to be impacted as a result of exposure to one or more environmental stressors, such as xenobiotics. Risk is assessed by comparing the exposure data with ecological effects to evaluate the likelihood of adverse impacts associated with exposure to a given stressor. Therefore, risk assessment depends on the environmental exposure of organisms to a certain contaminant, and its effects result from the degree of exposure [15].

Scientific interest in the potential toxic effects of NPs on living organisms has significantly grown in recent years [16], suggesting that these contaminants are potentially more harmful than MPs [11,17,18,19,20]. However, our understanding of NPs remains at early stages [21], and there is a paucity of data regarding both the environmental exposure and toxic effects of NPs on biota at realistic environmental concentrations [22,23]. This knowledge gap hampers the finalization of risk characterisation of NPs [12].

Even the risk of MPs is difficult to estimate owing to many knowledge gaps [20]. Indeed, although MPs have been studied for a longer time and much more data are available on them than NPs, only a few articles on the risk assessment of MPs have been published. For instance, the pioneering studies by Burns and Boxall [24] and Everaert et al. [25] have characterized the joint risk of MPs in freshwater and marine systems. More recently, the risks associated with MPs in freshwater and marine waters have been assessed separately [26,27]. Furthermore, Jung et al. [28] proposed a marine MP risk assessment method that considers differences in size and shape. The outcomes of these assessments highlight an acceptable risk associated with the presence of MPs in water compartments, whereas environmentally relevant concentrations of MPs in soil pose a considerable risk to soil biota [29].

### 1.4. Aims of the Review

The present review aims to provide a useful reference for better understanding the phenomenon of NP pollution in the environment. This manuscript not only aims to summarize and analyze the most recent studies on NPs, but also proposes a new tailored ERA procedure scheme. Since current NP risk assessments prioritize the identification of environmental exposure and relative effects on biota [12,20,22,23], we discuss and summarize the following three aspects of these priorities in the present review:

(a) Exposure assessment: detection of NPs in environmental samples

Developing suitable and reliable analytical methods for quantifying the environmental occurrences of NPs is pivotal. As reported by Besseling et al. [30], the detection techniques were not capable of identifying and quantifying NPs in environmental matrices until 2019, and research is rapidly progressing towards the development of novel methodologies suitable for these purposes [31]. A possible approach for the detection of NP exposure in the environment is the use of biomonitors, which can overcome some analytical limitations in NP extraction due to the potential of these contaminants to bioaccumulate in organisms [32]. From this perspective, different approaches have been developed to extract and quantify NPs from biological samples. This review summarizes progress in the detection of NPs in biological samples, detailing the current status of the characterisation of these contaminants in biological samples.

(b) Effects assessment: effects of NPs at the relevant environmental concentrations on biota

Assessing the possible modes of action of NPs and quantifying their adverse effects are some of the main knowledge gaps in ERAs. As previously mentioned, ecotoxicological studies on NPs should be performed at realistic environmental concentrations to determine effective thresholds for comparison with existing environmental levels. In this context, the present review analyses data on the adverse effects of NPs on biota at environmentally realistic concentrations (µg·L^−1^). Briefly, articles in literature are compared to summarize the different polymer types, sizes, concentrations, model organisms, and effects.

(c) Proposal for the harmonization of NP ERA

We provide suggestions and propose strategies for NP ERAs, highlighting the current knowledge gaps and critical points and emphasizing the priorities for harmonizing future studies.

## 2. Materials and Methods

Literature was searched using the key-words ‘nanoplastics’ combined with ‘detection’ and ‘biological samples’, ‘effect’, and ‘environmental concentration’ or ‘risk assessment’. The Minerva (University of Milan) and Prometeo (Milan-Bicocca University) datasets were used for this research, with data covering the timeframe until December 2021. Only peer-reviewed articles were included. As previously stated, there are no universal definitions of different range sizes. To harmonize the collected data, only studies reporting the analyses of NPs with dimensions within the range of 1–1000 nm were included. In the first section of this review, we focus on the detection of NPs in environmental samples. A total of 16 articles were selected: eight demonstrating the occurrence of NPs in abiotic matrices and remaining regarding the development of appropriate methodologies for NP extraction from biological samples. In the second section, we overview the effects of NPs on organisms. For this, a further criterion for literature selection was applied: the maximum concentration tested should be below 100 µg·L^−1^, representing an order of magnitude considered realistic in the environment. Finally, 62 articles were included.

## 3. Exposure Assessment: Detection of NPs in Environmental Samples

Very limited scientific literature (only eight papers) regarding the occurrence and concentration of NPs in the environment is available.

The detection of these contaminants in complex matrices is a real challenge. As such, the nanometric size of NPs renders the common extractive approaches applied for MPs unsuitable [33], and the heterogeneity of NP polymers makes the application of techniques that are typically used for engineered nanomaterials (ENMs) difficult [11]. Contrary to ENMs, which present a uniform composition owing to their intentional production as nanoparticles, NPs contain a mixture of different polymer types, sizes, and shapes, because they are derived from the unintentional fragmentation of larger plastic items dispersed in the environment. Moreover, the presence of a large variety of additives in the plastic materials of origin hinders the identification of polymer [11,33,34].

Currently, there are no straightforward methodologies for the extraction, identification, and quantification of NPs in the environment [35]. To the best of our knowledge, only eight studies have successfully extracted these contaminants from abiotic field samples. These studies have documented the occurrence of nanoscale plastic particles in seawater [36], rivers [37], snow [38,39], air [40], soil [41], sand [42], and tap water [43]. As shown in Table 2, only a few studies have simultaneously reported the concentration and relative polymeric types of NPs present in samples [37,38,43].

The measured environmental concentrations of NPs are extremely low (μg·L^−1^), posing significant analytical challenges for their detection in the environment. A possible strategy to overcome some of these limitations is to consider the use of biomonitoring. Bioindicators are living organisms that provide information on environmental quality [44]. More precisely, exposure indicators (e.g., bioaccumulation of xenobiotics in tissues) are considered useful tools to obtain data on environmental pollutants [45].

Owing to their capacity to accumulate nanosized particles, organisms may accumulate higher concentrations of NPs in the biological matrix than in other complex matrices [32]. Following uptake, NPs, especially the small ones (<100 nm), can penetrate the biological membranes [46] and potentially bioaccumulate in tissues or organs, leading to their transfer along trophic chains. This assumption is supported by experimental evidence [47]. Briefly, Zhou et al. [47] reported for the first time that the concentration of NPs in aquatic organisms is approximately 1 μg·g^−1^. Therefore, we believe that the use of biomonitoring offers significant advantages. Specifically, once the most suitable biomonitor is selected for different matrices, the extraction methodologies for different organisms only require optimization at the digestion step. As with any novel approach to a challenging issue, biomonitoring presents critical points. Assuming that NP concentration can be measured in biomonitor tissues, composition of the medium to which organisms are exposed must be estimated. For classical contaminants, the conventional approach is to achieve medium exposure through partition coefficients (e.g., K_ow_). However, the factors driving NP bioaccumulation and/or bioconcentration remain largely unknown. Therefore, the correct approach to calculating NP concentrations in the environmental matrix in which the organisms live remains unknown. Notwithstanding, a promising approach to model the mass balance of ingestion and loss processes of MPs has been recently reviewed [30], highlighting the possibility of modelling NP uptake and release in a similar manner. To calibrate and validate models, experimental determination of concentration is pivotal; consequently, a sound methodology is essential.

To the best of our knowledge, eight studies to date have proposed different methodologies to achieve this goal [32,47,48,49,50,51,52,53] (Table 3). All these studies utilized non-properly ‘environmental’ organisms, since all samples were collected from local markets [32,47,48] or directly from farms [49,50]. To test the efficiency of the proposed methodologies, tissue samples were spiked with nanoPS. Zhou et al. [47] verified the suitability of their method in *Tilapia* sp. and, as mentioned previously, successfully proved the environmental occurrence of NPs in three different species, at concentrations ranging from 0.09 to 0.78 μg·g^−1^.

The application of such methodologies can aid better comprehension of NP exposure in the environment. Nonetheless, the detection of NPs in biological samples is limited by certain analytical issues. From this perspective, a harmonized methodology of biological extraction is required to favor intercomparisons amongst studies. In the following sections, innovations reported in studies listed in Table 3 are elaborated, comparing different proposed extraction methods, and highlighting their strengths and critical points.

### 3.1. Digestion of Organic Matter in Biological Samples

Steps required for the extraction of NPs from biological samples can be summarized as follows: sampling, pre-treatment, digestion, preconcentration, separation, identification, and quantification [35,47].

Regarding sampling methods, please refer to a recently published review on this topic [54]. The complexity of the biological matrix makes the detection of NPs challenging. Plastic particles must be isolated and separated from the organic matter of biological samples, which could lead to interference in the subsequent steps of extraction. In this perspective, digestion is a key step which should be suitable to remove the organic matter, preserving, however, the properties and quantity of NPs [33,55]. Two different approaches are commonly adopted: chemical degradation and enzymatic digestion [16,35]. The first approach involves the use of different chemicals for acid (e.g., HCl and HNO_3_) or alkaline (e.g., NaOH and KOH) digestion, whilst the second utilizes specific enzymes (e.g., proteinase K, papain) that degrade the organic matrix. Chemical degradation enables efficient removal of the background matrix and is a cost-effective approach. However, acid treatment has recently been questioned because it tends to destroy and cause aggregation of NPs polymers [16,49]. Therefore, an alkaline approach, which is less invasive, is recommended [51]. Zhou et al. [47] obtained satisfactory results using this approach to digest the organic tissues of fish samples. When comparing the efficiency of two different alkaline reagents, tetramethylammonium hydroxide (TMAH) and sodium hydroxide (NaOH), TMAH showed a greater recovery without NP aggregation. In another recent study, Muhammad et al. [56] digested the gut and intestinal contents of insect larvae following the digestion protocol proposed by Zhou et al. [48].

Meanwhile, the enzymatic approach is considered a valid alternative to alkaline degradation. Although this approach incurs higher costs [54], it is non-destructive and avoids particle aggregation [57]. Correia and Loeschner [49] compared acid degradation (HNO_3_) with enzymatic digestion (proteinase K) in muscle tissues of fish samples and demonstrated that the former approach led to NP aggregation and subsequent hindrance in the extractive steps, whilst the latter could overcome these analytical issues. Furthermore, a recently proposed enzymatic digestion protocol [57] has shown satisfactory results for NPs extraction from biological matrices [32,52].

### 3.2. Separation, Identification, and Quantification of NPs in Biological Samples

Following digestion, it is necessary to isolate and separate particles in the nanometric size range; identify their effective sizes, shapes, and concentrations, and confirm the effective plastic nature of the extracted polymers through chemical characterisation. Simultaneous achievement of these results remains a huge challenge because of the lack of an efficient methodology for the extraction, identification, and quantification of NPs. The main steps adopted in the proposed methodologies for the detection of NPs in biological samples are summarized in Table 4.

#### 3.2.1. NP Precipitation

Recently, two different methodologies have been proposed to separate and isolate NPs present in biological samples [47,48] by exploiting the tendency of NPs to aggregate and co-precipitate with suspended materials. Typically, NPs tend to be dispersed in the medium, since they are dominated by Brownian motion; however, under specific conditions of pH and organic matter concentration, they associate with the material present in the solution, with their consequent sedimentation [48,58].

Zhou et al. [47] utilized the high binding affinity of NPs to proteins to extract them from biological samples through ethanol precipitation. The authors successfully validated the effectiveness of the proposed method by spiking the digested muscle tissue samples from *Tilapia* sp. with 2 μg·g^−1^ of PS and PMMA of different sizes (50, 100, and 500 nm) and obtained high recovery rates for all polymer sizes. Moreover, using pyrolysis–gas chromatography–mass spectrometry (py–GC–MS), they reported excellent limits of detections (LODs) (0.03 and 0.09 μg·g^−1^ for 100 nm PS and PMMA, respectively). Further, by adopting this methodology for environmental samples, the authors succeeded in detecting nanoPS (0.093–0.785 μg·g^−1^) in three different aquatic species.

Gao et al. [48] proposed a protocol based on coagulation–sedimentation extraction (CSE) between NPs and diatomite. By spiking the tissue samples from oysters and mice with nanoPS (70 nm), microPS (2 μm), and diatomite (7 μm), the authors successfully isolated and extracted NPs. As opposed to MPs, NPs tend to bind diatomite and precipitate upon centrifugation; thus, NPs can be isolated from MPs and the remaining suspended material with high recovery rates (95%). Finally, the authors chemically characterized the extracted particles using py–GC–MS and obtained an LOD of 0.012 μg·g^−1^.

#### 3.2.2. AF4 and Chip Trapping

Various studies have adopted an approach based on asymmetrical flow-field fractionation (AF4) to separate NPs from spiked biological samples [49,50,51,52]. AF4 allows the fractionation of nanoparticles based on their hydrodynamic size, which upon combination with detectors, such as diode array detector (DAD) and multiangle light scattering detector (MALS), provides additional information on particle size and concentration [31,35].

The first studies that adopted this approach in biological samples performed AF4-MALS to separate NPs from spiked digested fish tissue [49] and bird shell samples [50]. Luo et al. [51] recently proposed an AF4-DAD-MALS approach to separate and detect nanoPS (from 30 to 500 nm) in spiked blood samples of rats (*Rattus norvegicus*), demonstrating the feasibility of this methodology to separate and quantify nanoPS in biological samples, with satisfactory results for >100 nm PS.

In a recent study, Valsesia et al. [32] proposed a methodology for separating biological sample fractions with the same particle size. The authors spiked tunicate samples (*Ciona robusta*) with nanoPS (100 nm) and subjected them to AF4-DAD-MALS following enzymatic digestion. Subsequently, through ultrafiltration, the AF4-derived fractions were concentrated to a small volume and placed on a chip. Exploiting the tendency of nanoparticles to aggregate as the sample dries, NP clusters were grouped in a small area of the chip, achieving a sufficient signal for analysis using confocal Raman spectroscopy (CRM). By adopting this procedure, the authors succeeded in the chemical characterisation of nanoPS, combined with the estimation of NP concentration using scanning electron microscopy (SEM) coupled with an energy-dispersive X-ray (EDX) analyzer. The proposed method yields concentration data that are comparable to environmental levels.

In another study, Valsesia et al. [52] proposed a similar methodology that involves drying a sample on a chip with arrays of cavities of different sizes. Owing to the peculiar structure of the chip surface, single nanoparticles fall into the cavities according to their size, allowing the formation of small NP aggregates. The precise positioning of NPs of a certain size in the corresponding nanocavities enables their examination using SEM and spectroscopy (CRM). By testing the methodology on mollusk tissues (*Mytilus galloprovincialis*) exposed to 100 nm nanoPS, the authors succeeded in isolating nanoPS using a chip with 300 nm pores on the surface and finally characterized the isolated particles using CRM.

Moraz and Breider [53], improving the previously proposed methods [59,60], proposed a fluorescence-based approach for the detection of nanoPS in biological tissues. The method uses a fluorescent probe (a particular molecular rotor) that can conjugate with NPs. However, owing to the complexity of the investigated biological matrix (*Mytilus edulis* tissues), the authors did not obtain satisfactory results; nonetheless, the methodology presents the feasibility of detecting and quantifying NPs in biological samples.

### 3.3. Comparison Amongst Different Approaches

By comparing the methodologies presented in the literature, attempts can be made to fill this gap for successful extraction, identification, and quantification of NPs in biological samples.

The extraction of NPs from biological samples requires an efficient digestion step that allows for the removal of organic matter without damaging or causing the agglomeration of NPs. From this perspective, enzymes (e.g., papain) and alkaline reagents (e.g., TMAH) appear to be the most promising. Once digestion is performed, several methodologies can be adopted. Currently, however, no straightforward approach that allows simultaneous collection of data on concentrations and polymer characterisation is available.

The precipitation approach, followed by py–GC–MS, is relatively simple, yields an excellent LOD (e.g., 0.03 μg·g^−1^ [47]) and a high recovery rate (>85% [47,48]), and allows for the detection of small NPs (e.g., nanoPS 70 nm, [48]). However, although the application of py–GC–MS allows for covering the entire nanometric range, it is a destructive method that makes it impossible to simultaneously obtain information on the physical properties of individual particles [35]. Furthermore, because this methodology involves size-selective precipitation, handling real samples containing NPs of different sizes may be difficult. From this perspective, the use of AF4 as a separation step may be an effective alternative for obtaining fractions with NPs of the same size.

Furthermore, methodologies based on the evaporation of sample droplets on specific chip surfaces, followed by CRM analysis, are non-destructive. Using these methods, the number of particles can be directly counted through SEM and their polymeric type can be directly confirmed through Raman spectroscopy, with satisfactory LODs. However, these approaches require advanced instrumentation (e.g., functionalized chips) and additional analytical steps, such as an added purification step involving focused ion beam (FIB) [52], ultrafiltration [32], and EDX analysis, which may result in greater reproducibility.

Owing to the complexity of NP pollution, formulation of an extraction protocol for these contaminants from biological samples warrants effort. However, we believe that the scientific community is moving in the right direction, with significant progress being made towards better understanding the effective concentrations and levels of NPs in the environment.

## 4. Effect Assessment: Evidence from Laboratory Experiments

The number of ecotoxicological studies on the adverse effects of NPs has considerably increased in recent years. Whilst many of these studies have focused on aquatic biota, few studies have considered the effects on terrestrial organisms. Moreover, the currently available literature on the effects of NPs is limited to nanoPS. To determine the ecotoxicological risks of NPs, an assessment of exposure should be compared to an assessment of effect thresholds. Here, we present an overview of the effects of NPs at an individual level and at the lower levels of the biological structures and functions.

### 4.1. Lethal Effects on Aquatic Species

**Figure 3:**
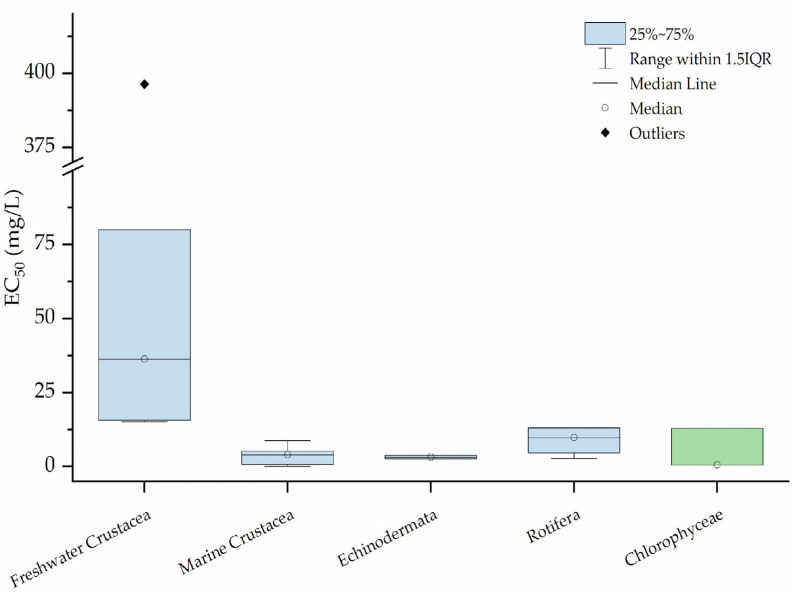
Lethal values (EC_50_) expressed in mg·L^−1^ of PS for different freshwater and marine taxa.

Only a few studies (11 articles to date [61,62,63,64,65,66,67,68,69,70,71]) have reported the classic toxicological endpoint of survival (EC_50_: effect concentration at which 50% of the exposed organisms are affected) following exposure to NPs, and these studies focused almost exclusively on PS, which is currently the most studied polymer. Of note, the exposure times vary amongst different tests, and often, the age of the tested organisms is not reported, particularly for *Daphnia* sp. This may increase the uncertainty and variability observed in the collected data (Figure 3). The concentrations of NPs affecting individual survival (EC_50_) are similar for organisms belonging to different taxa and/or phyla (Anostraca, Copepoda, Echinodermata, Rotifera, and Chlorophyceae) and are representative of diverse aquatic ecosystems (marine or freshwater). In particular, for marine crustaceans (*Artemia franciscana*, *Artemia salina*, and *Tigriopus japonicus*), echinoderms (*Paracentrotus lividus*), rotifers (*Brachionus plicatilis*), and chlorophytes (the marine alga *Dunaliella tertiolecta* and the freshwater alga *Pseudokirchneriella subcapitata*), EC_50_ values range from 0.54 to 13.0 mg·L^−1^. Conversely, higher values have been reported for freshwater crustaceans (*Daphnia magna*, *Daphnia pulex*, and *Macrobrachium nipponense*), with EC_50_ between 15.7 and 80 mg·L^−1^ for cladocerans and a much higher value of 396 mg·L^−1^ for decapods (*Macrobrachium nipponense*).

These results indicate that cladocerans are less sensitive to nanoPS than other taxa. This is particularly relevant because *Daphnia* sp. is used as a model organism in toxicological screening. From an ecological perspective, these results may provide unclear information on the sensitivity of natural zooplankton communities [72] and a proper assessment factor (AF) should be considered in risk characterisation to protect the entire aquatic community.

To describe the sensitivity of a given number of species to a xenobiotic, the statistical distribution function of species sensitivity distribution (SSD) is commonly used for assessing the hazardous concentration for 5% of the species (HC_5_) and safety concentration for 95% of the species in an ecosystem. Besseling et al. [30] built an SSD using both acute and chronic median lethal concentration (LC_50_), EC_50_, and the lowest observed effect concentration (LOEC) values available in the literature for aquatic organisms, derived an HC_5_ of 5.4 µg·L^−1^ for PS, and suggested applying an assessment factor of 5 in ERAs. However, this HC_5_ value involves several uncertainties and was extrapolated using limited available data obtained from marine, freshwater, and typical estuarine species.

Moreover, the LOEC values are usually considered controversial and inappropriate for this calculation because they can be influenced by study design [73,74]. Nevertheless, these values are currently acceptable because little data are available and SSD requires a specific number of points.

### 4.2. Sublethal Effects on Aquatic Species

NPs can penetrate biological membranes because of their small size, producing toxic effects at the cellular and molecular levels [75].

Amongst the studied organisms, the nematode *Caenorhabditis elegans* has emerged as a reliable toxicity model owing to its several practical and technical advantages [76]. We found 26 articles [77,78,79,80,81,82,83,84,85,86,87,88,89,90,91,92,93,94,95,96,97,98,99,100,101,102] reporting the sublethal effects of environmentally relevant concentrations (µg·L^−1^) of NPs on *Caenorhabditis elegans*. Of these, twenty-three studies examined the effects of nanoPS alone, whilst the other three considered the enhancement of the toxicity of other stressors in combination with exposure to nanoPS. However, the effects of NPs exposure in this taxon were limited to a few endpoints. Specifically, locomotion dysfunction was the most studied effect (17 of 23 articles). Many studies (16 of 23 articles) focused on reactive oxygen species (ROS) production to evaluate oxidative stress or intestinal damage. Almost all of these studies noted a decrease in locomotor activity and an increase in ROS production when *Caenorhabditis elegans* was exposed to an NP concentration of 1 µg·L^−1^ or higher. The only exception is the work of Zhao et al. [101], who noted the induction of these effects at concentrations exceeding 10 µg·L^−1^.

Five studies evaluated the potential of nanoPS to generate reproductive toxicity, whilst some studies focused on other molecular markers (i.e., neurotoxicity, metabolic toxicity, and so on).

Based on these observations, a concentration of 1 µg·L^−1^ can be assumed as a safety threshold for NPs to prevent the negative sublethal effects on wild organisms. Even at environmental concentrations (see Section 3) far below those that cause acute effects (see Section 4.1), continuous exposure to sublethal concentrations may lead to ecologically relevant adverse effects. Behaviour depends on integrated processes at the subcellular, cellular, and organismal levels and is thus susceptible to disruption by nanoPS. A change in locomotor activity can induce a series of indirect effects at the higher levels of ecological hierarchy (i.e., the community), such as alteration of the prey–predator interactions or competition for food and space [103,104].

To date, research on the effects of NPs on aquatic organisms was mainly focused on marine ecosystems. Studies on marine crustaceans (*Amphibalanus amphitrite* and *Artemia franciscana*) have demonstrated the negative effects of NPs on enzyme activities (acetylcholinesterase, propionyl cholinesterase, and catalase), indicating possible neurotoxicity and oxidative stress induction [105]. Moreover, negative effects of PS on catalase and glutathione S-transferase activities have been observed in fish cell lines [106]. Exposure to nanoPS showed adverse effects on the development of *Mytilus edulis* larvae [107] as well as induced oxidative damage and behavioural alterations and reduced regenerative capacity in *Hediste diversicolor* [108,109]. All of the above-mentioned effects were observed even after exposure of the organisms to low concentrations of PS (1 µg·L^−1^). Fish appear to be less sensitive, as behavioural alterations of locomotor activity, swimming hypoactivity, and bradycardia were observed in *Danio rerio* only after exposure to nanoPS concentrations as high as 100 µg·L^−1^ [110].

Amongst freshwater organisms, studies conducted on *Daphnia pulex* have demonstrated the induction of cytochrome P450 enzymes [111] and adverse effects on growth, development, and reproduction in organisms exposed to 100 µg·L^−1^ nanoPS [66]. Moreover, Liu et al. [112] reported that chronic exposure to 1 µg·L^−1^ nanoPS produced toxic effects on second-generation offspring, inducing stress and reducing growth rate and reproduction.

A few studies have examined the effects of amino-modified nanoPS. For instance, Balbi et al. [113] highlighted malformations of D-veligers in *Mytilus galloprovincialis* at environmental concentrations (from 1 µg·L^−1^) of nanoPS. Other laboratory experiments at higher nanoPS concentrations demonstrated changes in lipid composition and increases in lipid reserves in the marine alga *Chaetoceros neogracile* (50 µg·L^−1^). Further, effects on enzymatic activities and embryo-larval toxicity in marine crustaceans and mollusks exposed to nanoPS concentrations of 100 μg·L^−1^ or higher have been reported [114,115]. In contrast, only nanoPS concentrations exceeding 20 mg·L^−1^ have been shown to negatively affect the growth and biofilm formation of marine bacteria [116].

In addition to PS, only a limited number of studies have evaluated the effects of PMMA. For instance, Brandts et al. [117,118] reported increased expression of genes associated with lipid metabolism and antioxidant defense in marine fish exposed to environmentally realistic concentrations (1 µg·L^−1^) of PMMA. Booth et al. [119] observed significant toxicity in *Daphnia magna* exposed to poly(methyl methacrylate-co-stearyl methacrylate) copolymer, suggesting that surface chemistry plays an important role in ecotoxicity.

Of note, the majority of the toxicity studies used dialyzed particles without considering the presence of additives or other contaminants, which may pose more severe hazards to organisms than NPs themselves. Heinlaan et al. [120] assayed the toxicity of non-dialyzed nanoPS in *Daphnia magna*, *Raphidocelis subcapitata*, and *Vibrio fischeri* due to the presence of the biocidal additive NaN_3_ and other surfactants in the suspension.

The coexistence of NPs with other particles or chemical substances is crucial in NP research. Thanks to their specific surface area and hydrophobicity, NPs can, indeed, interact with other particles [121] and act as a vector of toxic molecules to the biota, causing the so-called ‘Trojan Horse’ effect [122]. There remain knowledge gaps regarding the possible combined effects of contaminants and NPs. However, two studies on *Caenorhabditis elegans* have demonstrated the potential role of nanoPS in enhancing the toxicity of other chemicals. First, Dong et al. [77] demonstrated that PS could enhance the toxicity of TiO_2_-NPs to decrease locomotor behaviour and induce intestinal ROS production. Second, Qu et al. [85] showed that nanoPS could increase the toxicity of the cyanobacterial toxin microcystin-LR to reduce brood size and locomotor behaviour and induce oxidative stress.

### 4.3. Lethal and Sublethal Effects on Soil Species

Soil constitutes an important reservoir of MPs and NPs [123]. The presence of plastic fragments has been recognized as one of the most important problems related to the quality of soil in agroecosystems [124], and the calculated risk for MPs has been assessed as unacceptable to soil biota [29]. Nevertheless, the problem of NP pollution in terrestrial environments has received less attention than in marine ecosystems. Identifying the effects and potential hazards on soil invertebrates is fundamental for preserving soil function. In addition to the nematode *Caenorhabditis elegans*, discussed in the previous section, which exhibits both aquatic and terrestrial free-living forms [125], few studies have investigated the effects of NPs on other terrestrial organisms.

As such, lethal effects on soil-dwelling organisms have been little investigated. Jiang et al. [126] observed significant changes in mortality in the earthworm *Eisenia fetida* exposed to NPs. Conversely, Zhu et al. [127] did not find any change in the mortality of soil oligochaete *Enchytraeus crypticus*.

Nevertheless, emerging evidence has confirmed the uptake of NPs and underscored their potential to cause detrimental effects following ingestion by soil organisms. Using realistic exposure experiments, Lahive et al. [128] verified the uptake of NPs by the earthworm *Lumbricus terrestris* and suggested the potential for long-term accumulation. The uptake of 100 nm PS was also confirmed in the earthworm *Eisenia fetida* after exposure for 14 days [126], with consequent histopathological changes in the intestinal tissue, induction of oxidative stress, and increase in the degree of DNA damage induced by low concentrations of PS (1 mg·kg^−1^ soil).

Furthermore, Zhu et al. [127] have highlighted the negative effects of NP exposure in soil organisms. The authors demonstrated a significant reduction in the weight and changes in the gut microbiome of the oligochaete *Enchytraeus crypticus* following exposure to 10% nanoPS (dry weight). However, they observed an increase in reproduction at lower NP concentrations.

Chae et al. [129] investigated the transfer of 28 nm PS particles from the soil to mung bean (*Vigna radiata*) plants and subsequently to snails (*Achatina fulica*) feeding on those plants in a terrestrial ecosystem. The authors noted a decrease in the root growth and nanoparticle accumulation in the leaves of mung bean plants. In addition, the authors highlighted a decrease in the growth rate as well as the feeding and foraging speeds of snails, which was associated with a decrease in the viability of gut microbiota and damage to the digestive tissues.

Additionally, the presence of additives used to produce plastics may damage soil organisms. Indeed, the biochemical and genetic toxicity of di-(2-ethylhexyl) phthalate (the most used additive in PVC production) has been demonstrated in the springtail *Folsomia candida* [130] and earthworm *Eisenia fetida* [64].

In the light of results reported by the above-mentioned studies, we cannot rule out the possible detrimental effects of NPs on terrestrial organisms and their potential transfer to organisms at higher trophic levels.

## 5. ERA Framework for NPs

Exposure to NPs is a major environmental concern. Based on evidence compiled to date, it can be emphasized that the measured nanoPS concentrations in river waters could produce sublethal effects in *Caenorhabditis elegans*. In fact, the concentrations (1.92–2.82 μg·L^−1^) measured by Zhou et al. [37] are higher than the proposed sublethal effective threshold of 1 μg·L^−1^, which has been proven to alter the locomotor ability and induce ROS production. The ubiquitous presence of NPs, their uptake by organisms, and their potential to act as vectors for toxicants and pathogens render their risk assessments a priority on stakeholders’ agendas at the global level. To the best of our knowledge, no framework for assessing the ecological risks of NPs has been adopted internationally. In principle, the current risk assessment paradigm for chemicals is applicable to nanomaterials and NPs. According to this classical scheme, the risk assessment process involves four steps: hazard identification, exposure assessment, effect assessment, and risk characterisation [30,131]. By combining the information on exposure levels (estimated or measured) with data on the expected effects, the final step of risk characterisation allows the establishment of whether the expected risk level could be considered acceptable. The risk quotient (RQ) is the ratio of potential exposure to a substance to the level at which no adverse effects are expected (RQ = exposure/toxicity). An RQ < 1 indicates an acceptable level of NP pollution, whereas an RQ  > 1 indicates a reason for concern. The current regulatory ERA is based on a tiered and mostly deterministic approach to rapidly identify the substances of low concern. The lower tiers present highly conservative assumptions and high levels of uncertainty; therefore, AF is used to cover all uncertainties in the approach. As such, AF decreases with each step of the tiered approach as more data are available, implying that the uncertainty in assessment decreases with each step [132]. Indeed, higher tiers are characterized by a higher degree of complexity but a higher ecological realism.

With the collected data, an exercise has been conducted to characterize the risk associated with the presence of nanoPS in the aquatic medium. In particular:

RQ_nanoPS_ = Exposure/Toxicity = 1.92–2.82 μg·L^−1^/(5.4 µg·L^−1^/5) = 1.7–2.6

where the exposure data are from Table 2; the toxicity data (HC_5_/AF) are from [30], and here above reported.

As the RQ is >1, it can be concluded that the current monitored concentrations of nanoPS in aquatic ecosystems pose an unacceptable risk for the wild communities.

The evidence of possible negative effects on both aquatic organisms and *Caenorhabditis elegans* highlights the urgent need to provide useful data for a sound risk assessment of nanoplastics in the environment.

**Figure 4:**
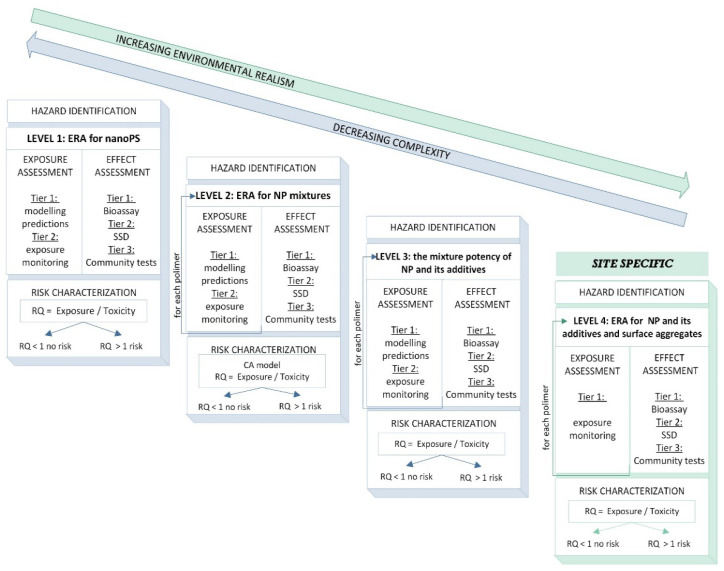
Tools for exposure and effect assessments as part of the general environmental risk assessment framework for NPs.

In this light, we suggest adopting the conventional frameworks proposed by Besseling et al. [30] and Koelmans et al. [131] with some addenda to realize a more realistic risk assessment scheme tailored to the specificities of NPs. The proposed method is illustrated in Figure 4.

We suggest a tiered framework in which four different levels of complexity are foreseen and characterized as follows:

### 5.1. Level 1: ERA for NanoPS

At this stage, we propose considering the conventional ERA scheme with its tiers, as detailed by Besseling et al. [30]. This step, which is less environmentally realistic but more easily applicable, is based on the assumption that the potential risk to wild organisms is generated by exposure to a single polymer. As highlighted in earlier, data on the effects of exposure to many polymers are scarce. Indeed, the majority of the studies to date focused on PS. Therefore, PS is suggested as a proxy for all NPs. To maximize the risk and simplify the ERA procedure, all fragments are assumed to be of the same size. However, the dimension that should be considered to better characterize the risk is not yet fully understood.

At this level of ERA, the estimated and/or measured concentrations of a type of polymer are compared with the safety thresholds extrapolated from the toxicological data of PS, that is, from a precautionary point of view, and the risk is calculated as follows:

$$\mathrm{RQ}=\frac{\overset{n}{\underset{i=1}{\sum }}\mathrm{EC}}{\mathrm{No}\mathrm{effect}-\mathrm{safety}\mathrm{threshold}\mathrm{for}\mathrm{PS}}$$

where i = different NP polymers.

We are aware that these assumptions are simple; however, they are internationally agreed upon in the risk assessment of poorly characterized contaminants. In some cases, owing to the lack of data on the effects induced by a chemical compound, data from a similar compound may be used, assuming that the effects can be assessed fairly well without the need for further testing. All assessments are based on the ‘read across’ principle, in which toxicological data related to chemicals, species, compartments, or endpoints are translated into data-free scenarios. Such approaches are convenient, albeit rather uncertain. Therefore, these methods should be placed at the lowest level of the ERA framework [133].

### 5.2. Level 2: ERA for NP Mixtures

As all produced polymers are released in the environment, to achieve greater ecological realism, the risk generated by an individual polymer should be calculated and then the overall mixture toxicity assessed. The potential mixtures likely to be present in the environment are virtually infinite; therefore, experimental testing is not feasible. In this context, predictive approaches for estimating the effects of mixtures with known compositions are required. The concentration addiction model [134] has been suggested as the acceptable worst-case precautionary approach [135]. According to CA, the toxicity of a mixture of NPs can be described as the sum of the so-called toxic units (TUs) of all mixture components. TU is the ratio of the concentration of a plastic polymer to the toxicological endpoint (EC_50_).

The mathematical CA concept for a multi-compound mixture can be expressed as follows:

$$\mathrm{Mixture}\mathrm{toxicity}=\overset{n}{\underset{i=2}{\sum }}\frac{\mathrm{ECi}}{{\mathrm{ECx}}_{i}}$$

where n is the number of compounds in the mixture causing x% of the total effect; Ci is the concentration of the ith component; and ECx_i_ is the concentration of the respective component that produces the same effect when applied individually.

Although CA is based on the assumption that all components of a mixture have the same mode of action, this concept also enables reliable assessment of the effects of mixtures of non-similarly acting chemicals.

### 5.3. Level 3. Mixture Potency of NPs and Their Additives

Plastics used in commercial goods are not composed of pure polymers and often contain organic additives and inorganic co-formulants in their mixture. In the light of evidence that co-formulants can enhance the effects of nude NPs or even act as sinergizants [120], the risk characterisation of plastic mixtures is essential. ERA should be performed with toxicological endpoints obtained by testing the true composition of plastics, that is, nude NPs and their additives.

### 5.4. Level 4: ERA for NPs with Their Additives and Surface Aggregates

In the environment, there exist types of plastics that differ not only in chemical composition, shape, and size but also in the composition of surface aggregates. Numerous environmental pollutants and microbial communities colonize NP surfaces. ERA at this level is extremely complex and feasible only with exposure data and site-specific effects. Level 4 should be applied in specific case studies related to a certain environmental reality, in which a high degree of knowledge and characterisation of NP contamination is required.

## 6. Conclusions and Research Recommendations

Data for robust risk characterisation are not available; however, with evidence accumulated to date, it can be emphasized that environmental concentrations of nanoPS induce unacceptable effects in diverse wild organisms. In this context, we propose a methodology for the ERA of NPs based on different tiers characterized by ever-increasing, deepening levels, complexity, and ecological realism. However, there remain substantial knowledge gaps related to NP exposure and toxicity fields, which hamper the finalization of ERA. Moreover, there is an urgent need for harmonization between different researchers and stakeholders both in terms of techniques and concepts. Firstly, the univocal definition of NPs is strongly suggested.

In this light, we therefore suggest the following recommendations for the future research:

(1) An important aspect is the complexity of establishing an optimal analytical method for identifying and quantifying NPs in environmental matrices owing to their extremely low concentrations. The use of a biomonitor is a reliable strategy to overcome these limitations, although predicting NP concentrations in the environments in which these organisms live based on bioaccumulated levels remains a critical point. We therefore suggest applying the above reported methodologies on organisms belonging to different trophic levels, especially of terrestrial ecosystems.
(2) We support the need for an harmonization of the experimental procedures in NPs ecotoxicology studies. NPs often contain different additives that can leach out either in the exposure media or in the intestine after being ingested, which might lead to the increased toxicity to the studied organism. Cleaning the NPs suspension from additives is a fundamental step. Nevertheless, there are still no clear guidelines on how to clean these suspensions, and this involves issues of reproducibility and comparability among the different studies. Moreover, since it is difficult to obtain information about co-formulants included in marketed plastics, it would be beneficial if plastic producers would provide this information.
(3) The influence of size on the efficiency of internalisation and on toxicity is still scarcely investigated. Therefore, a better characterization of toxicity in the function of different particle sizes should be provided for a more comprehensive understanding of most hazardous particle dimensions.
(4) Another important knowledge gap is related to the toxicities of different polymers; indeed, information is mostly limited to nanoPS, whilst other NPs await further work.
(5) In addition, a limited number of ecotoxicological studies have been performed at realistic environmental concentrations.
(6) Furthermore, a vast majority of studies have focused on aquatic biota, although NP pollution extends to terrestrial environments, such as agroecosystems. These represent largely under-investigated sources of NP contamination, and the potential impact of NP pollution on terrestrial biota warrants thorough investigation.

The foreseen increase in environmental NP concentrations underscores the need for the investigation of their impacts on wildlife by both the scientific community and regulatory bodies.

## Figures and Tables

**Table 1:** European plastic demand in 2020. The column ‘Mt’ presents the annual demand of plastic expressed in million tons (total: 49.1 Mt). For further details, please refer to [5]. EPS: expandable polystyrene; PMMA: polymethylmethacrylate.

Plastic Polymer	Mt	%	End-Use Market
PE	14.9	30.3	Food packaging, bulding, construction
PP	9.7	19.7	Food packaging, automotive
PVC	4.7	9.6	Bulding, construction
PET	4.1	8.4	Food packaging
PUR	3.8	7.8	Bulding, construction, others
PS/EPS	3.0	6.1	Food packaging, bulding, construction
Other plastics (e.g., epoxy resins, PMMA)	8.9	18.1	Bulding, construction, automotive, others

**Table 2:** List of the studies that successfully detected NPs in environmental abiotic matrices (updated to December 2021). PA: polyamide; PO: polyolefins.

Samples	Concentration	Polymer Type	References
Seawater	-	PET, PS, PE, PVC	[36]
River water	1.92–2.82 μg/L	PS	[37]
Snow	46.5 μg/L	PET, PP	[38,39]
Air	-	PET, PS	[40]
Soil	-	PVC, PS, PE	[41]
Sand	-	PVC, PS	[42]
Tap water	1.67–2.08 μg/L	PVC, PS, PA, PO	[43]

**Table 3:** List of studies that developed novel methodologies for the detection of NPs in biological samples.

Organism	Target	Polymer	Size (nm)	Ref.
Mollusk, Crustacean, Fish	Muscle	PS, PMMA	100	[47]
Mollusk (oyster) Mammal (C57BL/6 mice)	Whole organism Gut, liver, kidney	PS	70 70	[48]
Fish (*D. labrax*)	Muscle	PS	100	[49]
Bird (non-specified)	Eggshell	PS	60, 200, 600	[50]
Mammal (*R. norvegicus*)	Blood	PS	100, 200, 500	[51]
Tunicate (*R. ciona*)	Whole organism	PS	100	[32]
Mollusk (*M. galloprovincialis*)	Whole organism	PS	100, 500, 1000	[52]
Mollusk (*M. edulis*)	Stomach	PS	49	[53]

**Table 4:** Methods for the detection of NPs in biological samples. TEM: transmission electron microscopy; SEM: scanning electron microscopy; py–GC–MS: pyrolysis–gas chromatography–mass spectrometry; CSE, coagulation-sedimentation extraction; AF4, asymmetrical flow-field fractionation; DAD, diode array detector; MALS, multiangle light scattering detector; CRM, confocal Raman spectroscopy; EDX, energy-dispersive X-ray; FIB, focused ion beam.

Digestion	Separation	Quantification	Identification	Ref.
Alkali (TMAH)	Ethanol-precipitation	TEM	py-GC-MS	[47]
Alkali (KOH)	CSE	-	py-GC-MS	[48]
Enzimatic (Proteinase K)	AF4	AF4-MALS	-	[49]
Acid (HCl) Alkali (TMAH)	AF4 Ultracentrifugation	AF4-MALS	-	[50]
Alkali (KOH)	AF4	AF4-DAD-MALS TEM	-	[51]
Enzimatic (Papain)	AF4, Ultrafiltration Chip trapping	AF4-DAD-MALS SEM	CRM SEM-EDX	[32]
Enzimatic (Papain) FIB	Microcavity-size selection	SEM	CRM SEM-EDX	[52]
Alkali (NaOH, KOH)	Centrifugation	Microplate fluorescence reader	-	[53]
